# Femoral anteversion: significance and measurement

**DOI:** 10.1111/joa.13249

**Published:** 2020-06-24

**Authors:** Matteo Scorcelletti, Neil D. Reeves, Jörn Rittweger, Alex Ireland

**Affiliations:** ^1^ Department of Life Sciences Research Centre for Musculoskeletal Science & Sports Medicine Manchester Metropolitan University Manchester UK; ^2^ Institute of Aerospace Medicine German Aerospace Center (DLR) Cologne Germany; ^3^ Department of Paediatrics and Adolescent Medicine University of Cologne Cologne Germany

**Keywords:** antetorsion, hip, joint shape, proximal femur, skeletal development

## Abstract

Femoral neck anteversion (FNA) is the angle between the femoral neck and femoral shaft, indicating the degree of torsion of the femur. Differences in FNA affect the biomechanics of the hip, through alterations in factors such as moment arm lengths and joint loading. Altered gait associated with differences in FNA may also contribute to the development of a wide range of skeletal disorders including osteoarthritis. FNA varies by up to 30° within apparently healthy adults. FNA increases substantially during gestation and thereafter decreases steadily until maturity. There is some evidence of a further decrease at a much lower rate during adulthood into old age, but the mechanisms behind it have never been studied. Development of FNA appears to be strongly influenced by mechanical forces experienced during everyday movements. This is evidenced by large differences in FNA in groups where movement is impaired, such as children born breech or individuals with neuromuscular conditions such as cerebral palsy. Several methods can be used to assess FNA, which may yield different values by up to 20° in the same participant. While MRI and CT are used clinically, limitations such as their cost, scanning time and exposure to ionising radiation limit their applicability in longitudinal and population studies, particularly in children. More broadly, applicable measures such as ultrasound and functional tests exist, but they are limited by poor reliability and validity. These issues highlight the need for a valid and reliable universally accepted method. Treatment for clinically problematic FNA is usually de‐rotational osteotomy; passive, non‐operative methods do not have any effect. Despite observational evidence for the effects of physical activity on FNA development, the efficacy of targeted physical activity remains unexplored. The aim of this review is to describe the biomechanical and clinical consequences of FNA, factors influencing FNA and the strengths and weaknesses of different methods used to assess FNA.

## Graphical abstract

Femoral neck anteversion (FNA) is the angle between the femoral neck and femoral shaft, which affects the biomechanics of the hip. FNA changes substantially throughout growth, which may relate to motor development, and varies by up to 30° within adults. Several methods can be used to assess FNA, which may yield different values by up to 20° in the same participant. Treatment for clinically problematic FNA is usually de‐rotational osteotomy; passive, non‐operative methods do not have any effect.

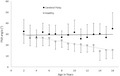

## 1 OVERVIEW

**FIGURE 1:**
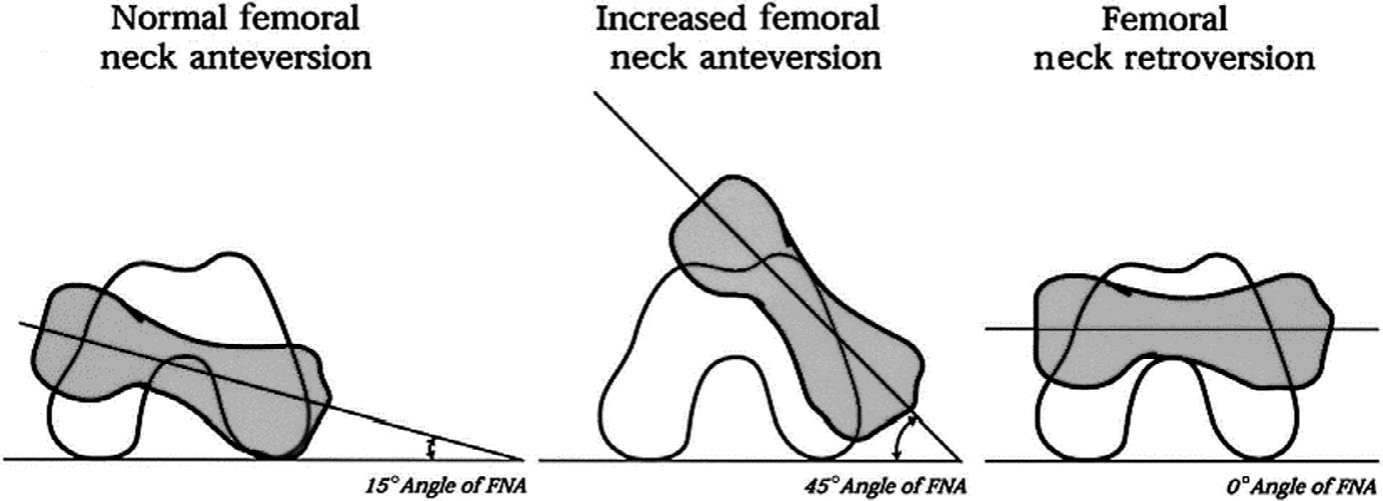
Axial schematic representation of the right femur and of the femoral neck anteversion (FNA). The grey area represents the femoral neck and the white area represents the distal condylar region. From Cibulka (2004)

Femoral neck anteversion (FNA), also called femoral torsion or femoral version, is the angle between the projection of two lines in the axial plane perpendicular to the femoral shaft; one line going through the proximal femoral neck region and the second one through the distal condylar region (Figure 1), indicating the degree of ‘twist’ of the femur. FNA affects the biomechanics of the hip, as moment arms and the line of action of muscles around the joint are altered. As a result, FNA is associated with differences in gait and is a risk factor for clinical problems including osteoarthritis and slipped capital femoral epiphysis. FNA goes through substantial development during growth with a change from 0° in early gestation to 30° at birth, decreasing to 15° in adulthood. In addition to age, FNA appears to be strongly affected by mechanical loading during movement, such that several clinical conditions associated with delayed or impaired locomotion are associated with greater FNA.

There are several methods to assess FNA, including imaging using radiography, fluoroscopy, computed tomography (CT), ultrasound (US), and magnetic resonance imaging (MRI) as well as functional assessments. Even within each imaging method, there are variations in how anatomical landmarks are identified. Differences in cost, time, availability, repeatability and radiation exposure mean that certain methods are not applicable, e.g. for clinical studies or those involving children.

The aim of this review is to discuss the implications of altered FNA, in terms of both its effects on movement and its clinical consequences. In addition, we describe normal variation and factors affecting FNA in healthy and clinical populations of different ages. Finally, we will outline the different methods and landmarks used to assess FNA and evaluate their strengths and weaknesses with regard to a defined study setting.

## 2 BIOMECHANICAL SIGNIFICANCE OF FNA

**FIGURE 2:**
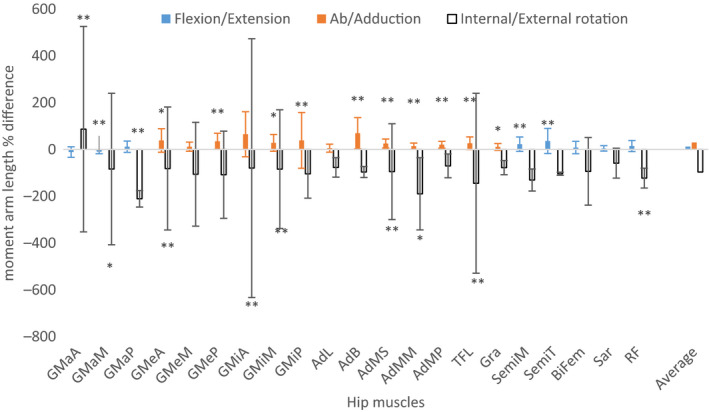
Effects of altered femoral neck anteversion (FNA) on reference moment arm length (MAL), calculated as the difference between subject‐specific model and a general model. The subject‐specific model is taken as the reference and therefore negative values indicate a higher value in subject‐specific models and vice versa. The subject‐specific model is an average of subjects with a high FNA (ranging from 25 to 51). The moment arm length is averaged over the whole range of motion (10° extension 90° flexion, 50° abduction, 20° adduction, 40° external and 40° internal rotation). Only the main function is recorded for every muscle and the internal/external rotation. Conventionally positive values are used for flexion abduction and internal rotation. Figure created using data from Scheys et al. (2008). GMaA, gluteus maximus anterior; GMaM; gluteus maximus medialis; GMaP Gluteus maximus posterior; GMeA, gluteus medius anterior; GMeM, gluteus medius medialis; GMeP, Gluteus medius posterior; GMiA, gluteus minimus anterior; GMiM, Gluteus minimus posterior; AdL, adductor longus; AdB, adductor brevis; AdMS, adductor magnus superior; AdMM, adductor magnus middle; AdMP, adductor magnus inferior; TFL, tensor fascia latae; Gra, gracilis; SemiM, semimembranosus; SemiT, semitendinosus; BiFem, biceps femoris long head; Sar, sartorius; RF, rectus femoris. Asterisks denote statistical significance:: *p < .05 and **p < .01

A change in FNA affects the position of the trochanter and therefore the line of action of the muscles surrounding that region. Regional torsional changes along the femur also result in a change of lever arms (Kim *et al.,* 2012). A higher FNA results in a slightly shorter hip extension moment arm and an increase in hip flexion moment arm of the abductor muscles. Furthermore, high FNA results in a shorter abductor lever arm (Scheys *et al.,* 2008; Li *et al.,* 2014) and also considerably increases internal rotation moment length by an average of 96.5% for all hip muscles (Figure 2), apart from the iliopsoas, which was not evaluated, and the gluteus maximus anterior, which decreases internal rotation moment arm length by 86% (Scheys *et al.,* 2008). A higher FNA also affects muscle activation, as lower gluteus medius and vastus medialis activity has been recorded during isometric hip abduction (Nyland *et al.,* 2004) probably due to the change in moment arm length. The higher FNA, and therefore the shorter abductor lever arm, also changes the mechanics of the hip joint resulting in up to 24% higher hip contact forces during gait with an anteversion of 30° and 8% higher forces with FNA of 14°, when compared with an anteversion of −2° (Heller *et al.,* 2001; Li *et al.,* 2014). On the other hand, reduced FNA results in higher shear forces on the femoral neck‐head junction (Pritchett and Perdue, 1988), quantifiable as a 42% increase with an FNA of 0° and 86% with an FNA of −12.5° and 12.5°, respectively (Fishkin *et al.,* 2006). Distally, increased FNA is associated with a progressive increase in patellofemoral contact pressures (Lee *et al*., 1994; Lee *et al*., 2003).

## 3 CONSEQUENCES OF ALTERED FNA FOR HEALTH

Altered movement associated with differences in FNA also appears to have consequences for musculoskeletal health. The shorter hip extension moment arm and longer moment arm for hip flexion shown with increased FNA are consistent with the gait pattern in individuals with cerebral palsy, and the shorter abductor and adductor lever arms are likely to produce pelvic instability during gait (Laplaza and Root, 1994; Scheys *et al.,* 2008). The increased internal rotation moment arm length, combined with a decreased external rotation moment arm length, is likely to be part of the cause of in‐toeing gait in children with cerebral palsy (Gelberman *et al.,* 1987; Fabry *et al.,* 1994; Scheys *et al.,* 2008; Uemura *et al.,* 2018) as a strategy to increase the abductor lever arm during movement (Arnold *et al.,* 1997; Uemura *et al.,* 2018). Self‐adjustment of in‐toeing gait is often accompanied by the compensatory external rotation of the tibia (Fabry *et al.,* 1973). Greater FNA is also associated with a number of orthopaedic pathologies (Gulan *et al.,* 2000), including increased risk of anterior cruciate ligament injury (Nyland *et al.,* 2004; Shultz *et al.,* 2008; Amraee *et al.,* 2017), which might be related to altered knee kinematics during landing (Howard *et al.,* 2011), lower hip abductor and vastus medialis activity (Nyland *et al.,* 2004), impaired tracking of the patella (Reikerås, 1992; Lee *et al.,* 1994; Seitlinger *et al.,* 2016; Kaiser *et al.,* 2017; Imhoff *et al.,* 2019) and femoral trochlear dysplasia (Liebensteiner *et al.,* 2016). Greater hip load and the altered relationship with the acetabulum––resulting from increased FNA (Reikerås *et al.,* 1983)––may play a role in the genesis of osteoarthritis (McSweeny, 1971; Reikerås and Høiseth, 1982; Li *et al.,* 2014; Fujishiro *et al.,* 2014; Inamdar *et al.,* 2019). This suggestion is reinforced by a prevalence of unilateral osteoarthritis in limbs with higher FNA (Halpern *et al.,* 1979; Piazzolla *et al.,* 2018). The decreased congruity could also result in hip dysplasia, a condition that displays FNA averages of 6°– 18° above normal (Alvik, 1962; Fabry *et al.,* 1973; Anda *et al.,* 1991; Sugano *et al.,* 1998b; Li *et al.,* 2014; Lerch *et al.,* 2018), whereas hip congruity (Reikerås *et al.,* 1983) and loading (Heller *et al.,* 2001; Satpathy *et al.,* 2015) might be a contributors to femoral acetabular impingement (Sutter *et al.,* 2015; Chadayammuri *et al.,* 2016; Gómez‐Hoyos *et al.,* 2016; Lerch *et al.,* 2018). On the other hand, the aforementioned increased shear forces occurring with reduced FNA could explain the association of slipped capital femoral epiphysis with populations which have low FNA (Gelberman *et al.,* 1986). Not only is increased or decreased FNA a risk factor for clinical conditions, but asymmetries in FNA also appear to influence musculoskeletal health, as shown by Piazzolla *et al*. (2018). In this study, patients with unilateral osteoarthritis of the hip with higher anteversion reported lower back pain, whereas unilateral osteoarthritic subjects with symmetrical FNA did not. FNA has been shown to affect the accuracy of clinically relevant bone mineral density measures (Cheng *et al.,* 1997). However, little is known about whether FNA and altered biomechanics could also affect bone mineral density, other bone strength indicators or the risk of femoral fractures through these factors or by altered fall mechanics.

## 4 EPIDEMIOLOGY

**FIGURE 3:**
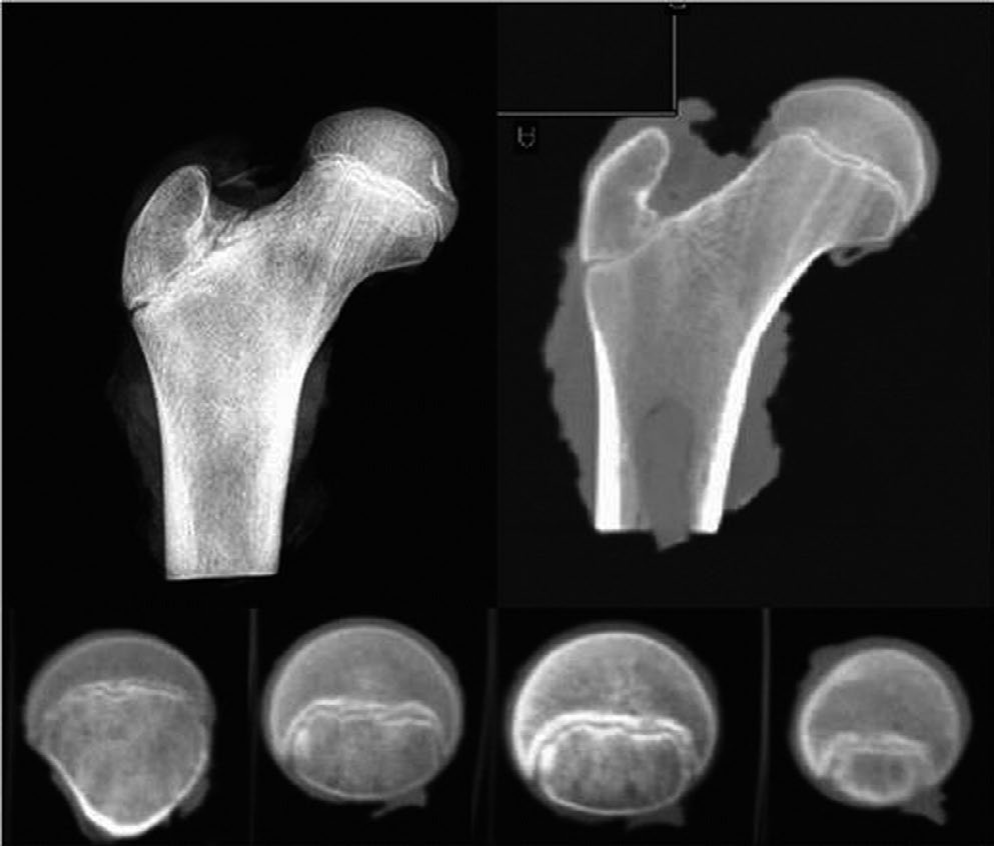
Epiphyseal growth plate: Radiography and computer tomography (CT) of cadaveric proximal femur of 13‐year‐old individual. Coronal view on top panels, axial view in bottom panels (Kandzierski *et al.,* 2012)

Normative data for FNA in the healthy adult population is highly dependent on the landmarks identified and imaging technique used (Kaiser *et al.,* 2016), with mean values in the range of 7°–24° (Starker *et al.,* 1998; Sugano *et al.,* 1998a; Kuo *et al.,* 2003; Toogood *et al.,* 2009; Botser *et al.,* 2012; Sutter *et al.,* 2015; Lerch *et al.,* 2018). In addition, there is substantial variation within the population, with individual values ranging by more than 30°, independent of the method used (Yoshioka *et al.,* 1987; Waidelich *et al.,* 1992; Toogood *et al.,* 2009; Sangeux *et al.,* 2014; Rosskopf *et al.,* 2014). FNA is at least partly hereditary, with a polygenetic influence on this and other features of proximal femur shape (Hogervorst *et al.,* 2012). However, another key factor is the influence of mechanical loading during everyday movements and exercise. Both the greater trochanteric and the epiphyseal growth plate (Figure 3) are accountable for shaping the proximal femur (Fabeck *et al.,* 2002). Bone growth has been shown to be directed perpendicularly to the direction of the growth plate (Dallek and Jungbluth, 1984; Hunziker, 1994), which is orientated in line with the forces acting on it (Pauwels and Maquet, 1979; Carter *et al*., 1987; Fabeck *et al*., 2002). The growth rate of growth plate cartilage is influenced by mechanical loading, such that increased compressive and tensile loading increases growth rate up to a point, with additional loading leading to reduced growth rate and potential damage (Rauch, 2005).

**FIGURE 4:**
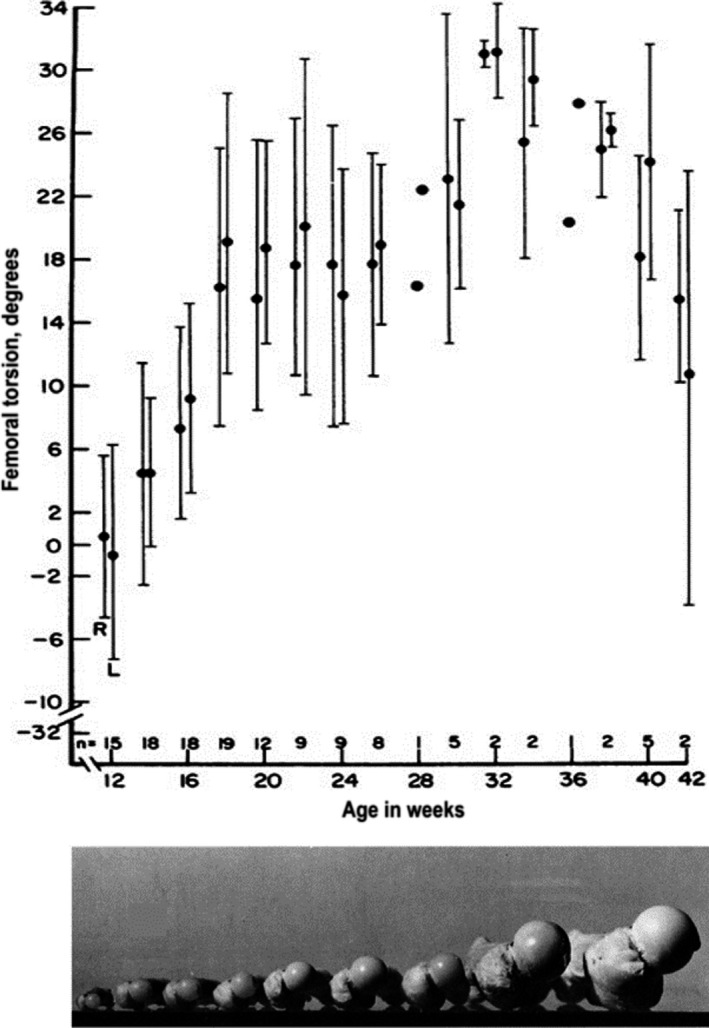
Femoral neck anteversion (FNA) means and standard deviation of fetuses at different stages of gestation. Bottom panel shows photos of typical fetal femur samples at different developmental stages (12 weeks to term). Figures adapted from Walker and Goldsmith (1981)

**FIGURE 5:**
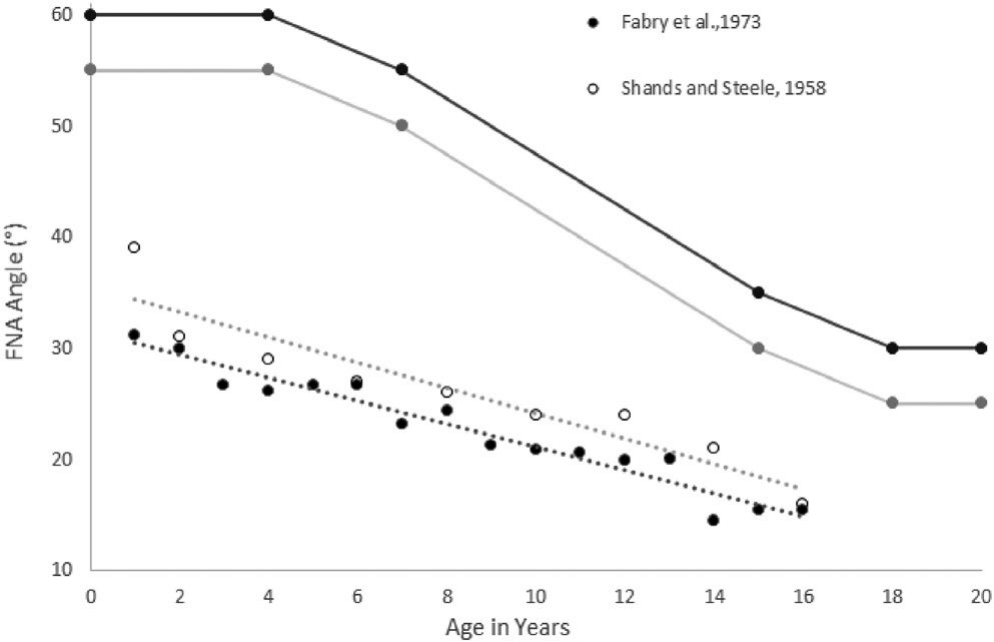
Mean values and normal/pathological limits of femoral neck anteversion (described as antetorsion or AT angle) in children of different ages as measured by different investigators (Shands and Steele, 1958; Fabry *et al.,* 1973; Tönnis and Heinecke, 1991)

This effect of mechanical loading likely contributes to the dramatic changes in FNA observed throughout prenatal development and childhood. An increase of around 30° in FNA during fetal life has been observed (Figure 4) (Watanabe, 1974; Walker and Goldsmith, 1981; Jouve *et al.,* 2005; Li *et al.,* 2019), particularly during the second trimester. In the womb, the hip has a high angle of flexion and the femur is levered against the antero‐superior iliac spine, thereby increasing the torsional strain favouring anteversion (Hogervorst *et al.,* 2012). The internally rotated position of the hip joint during fetal life could also result in increased anteversion, and the opposite is true for external rotation (Watanabe, 1974). This was confirmed in animal studies with forced internal rotation (Wilkinson, 1962) and by the 10° higher FNA found in children born with breech presentation (Hinderaker *et al.,* 1994); these children often have an internally rotated position in the womb resulting in reduced kicking forces and lower femoral stress and strain during fetal movements (Verbruggen *et al.,* 2018). During childhood, a steady ~1.5° a year decrease in anteversion until completion of growth has been recorded (Figure 5) (Fabry *et al.,* 1973; Svenningsen *et al.,* 1989; Tönnis and Heinecke, 1991). This decrease during growth might depend on the action of hip muscles during gait, which may shape the FNA (Yadav *et al.,* 2017) and keep the resultant forces during the maximal weight‐bearing period perpendicular to the growth plate (Fabeck *et al*., 2002).

There is some evidence from large cohort cross‐sectional studies to suggest that FNA also decreases, at a lower rate, during adulthood (Waisbrod *et al.,* 2017; Pierrepont *et al.,* 2019). This raises the question of whether 50–70 years ago children were more active, and whether we are observing secular rather than within‐individual changes. Furthermore, a recent longitudinal study in individuals with hip osteoarthritis suggests that FNA decreases with time over a period of 3 years (Inamdar *et al.,* 2019). This might be due to localised addition of bone on the periosteal surface with increasing age, microfractures or the bony erosion due to the osteoarthritic condition. Most studies show a higher FNA in the female population with sex differences ranging from 2° to 8° (Fabry *et al.,* 1973; Cyvín, 1977; Bråten *et al.,* 1992; Tamari *et al.,* 2006; Decker *et al.,* 2013; Fujishiro *et al.,* 2014; Sutter *et al.,* 2015; Chadayammuri *et al.,* 2016; Lerch *et al.,* 2018). It is known that growth plate fusion occurs at an earlier age in women than men (Grumbach, 1992), therefore a shorter growth period could be a cause of the higher FNA in women. Although FNA values reported in individuals from different ethnic groups have differed substantially, this may relate to the use of different measurement methods. More recent CT studies have found no differences in FNA based on ethnicity (Koerner *et al.,* 2013).

**FIGURE 6:**
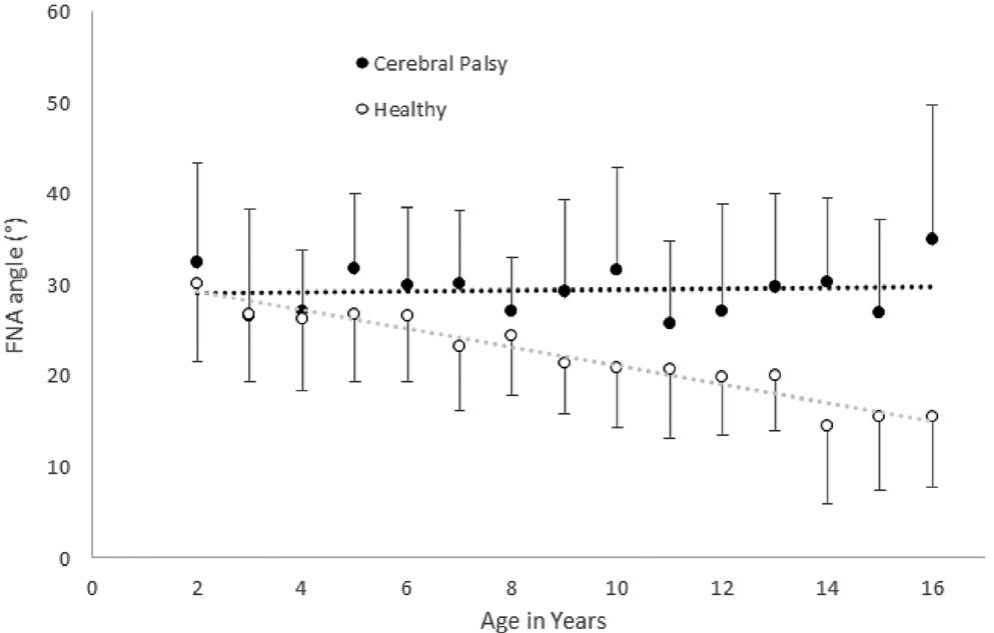
Femoral neck anteversion (FNA) in children with cerebral palsy (CP) and typically developing controls during growth, presented as mean and standard deviation. Adapted from Bobroff *et al*., 1999 (Bobroff *et al*., 1999)

The importance of mechanical loading for FNA during development is also evident from altered values in children with compromised motor development and movement. Notably, children with CP do not show a decrease in FNA during development (Figure 6) (Fabry *et al.,* 1973; Bobroff *et al.,* 1999). Typically, FNA is around 10° higher in older children with CP than unaffected children, with similar differences evident between the affected and unaffected limbs in children with hemiparetic CP (Staheli *et al.,* 1968). This is thought to be caused by spasticity or decreased activation of certain muscle groups, which is frequent in the clinical spectrum of CP subjects. In particular, it was suggested that increased activity of adductor and extensor muscles predicts higher FNA, as does reduced activity of hip flexors (Yadav *et al.,* 2017). This was confirmed in animal studies resecting either internal or external rotator muscles (Haike, 1964). Interestingly, ambulant children with CP have higher FNA than non‐walking children with CP (Bobroff *et al.,* 1999). This suggests that the alterations in muscle activity and subsequent joint loading during gait in children with CP contribute to development of FNA and therefore that healthy motor development is an important factor in development of the proximal part of the femur (Yadav *et al.,* 2017). This is supported by reports suggesting that differences in FNA between children with CP and normally developing children emerge at around 12 months, the typical onset of independent walking (Beals, 1969).

FNA has also been reported to differ from typical values in children with other conditions affecting neuromuscular development, such as Down syndrome, with an average of 33° (Shaw and Beals, 1992) and Charcot‐Marie‐Tooth disease, where the mean FNA is 28° (Novais *et al.,* 2014). In addition, higher values are observed in children with a range of disorders affecting skeletal development. For example, Blount's disease causing bowing of the tibia (Aird *et al.,* 2009), Legg‐Calvé‐Perthes disease resulting in avascular necrosis of the femoral head (Lerch *et al.,* 2018) and achondroplasia (Song *et al.,* 2006), which results in substantially reduced limb length. On the other hand, obesity in adolescence is associated with an FNA of only 0.4° ± 13° (Galbraith *et al.,* 1987). This could be due to the increased muscular forces required to move a greater body mass during development.

(Kandzierski *et al.,* 2012).

## 5 TREATMENT

Idiopathic altered anteversion in early childhood usually corrects itself without intervention (Fabry *et al.,* 1973; Svenningsen *et al.,* 1989; Staheli, 1993). In cases where increased FNA does not correct itself and results in in‐toeing and tripping, the most effective method to change FNA is femoral de‐rotational osteotomy. Clinical considerations such as indications, imaging, surgical techniques and associated results, and anatomical considerations have been reviewed in detail by Nelitz (2018) and are discussed only briefly here. De‐rotational osteotomy can be performed either at a distal supracondylar level (Hoffer *et al.,* 1981) or at a proximal sub‐trochanteric or intertrochanteric level (Payne and Deluca, 1994). The contribution of these different regions to total anteversion differs within and between clinical groups (Kim *et al.,* 2012; Seitlinger *et al.,* 2016). Therefore it has been suggested that the planning of osteotomies should take into account this segmental variation in order to ensure healthy postoperative hip biomechanics and prevent further clinical problems (Kim *et al.,* 2012; Ferlic *et al.,* 2018). De‐rotational osteotomy has been shown to be a successful technique in the treatment of in‐toeing children with cerebral palsy (Saglam *et al.,* 2016; Sung *et al.,* 2018) and patellar instability (Nelitz *et al.,* 2015; Imhoff *et al.,* 2019). However, it must be taken into account that complications might arise and the whole recovery process could be a traumatic experience (Staheli, 1993). Therefore, surgery is suggested only in disabling or symptomatic instances, only after the age of 10 and, depending on the condition, in cases of a measured anteversion above 20°–50° (Staheli, 1993; Nelitz *et al.,* 2015; Weber *et al.,* 2016; Nelitz, 2018) and internal rotation higher than 80° (Staheli, 1993; Leonardi *et al.,* 2014). Non‐operative methods to lower FNA such as shoe wedges, twister cables and night splints have been proposed but do not appear to be effective (Fabry *et al.,* 1973; Knittel and Staheli, 1976). The effects of movement and motor development on FNA described earlier suggest that physical therapies and targeted exercises may alter FNA during growth, but to our knowledge this remains unexplored.

## 6 FEMORAL AXES

**FIGURE 7:**
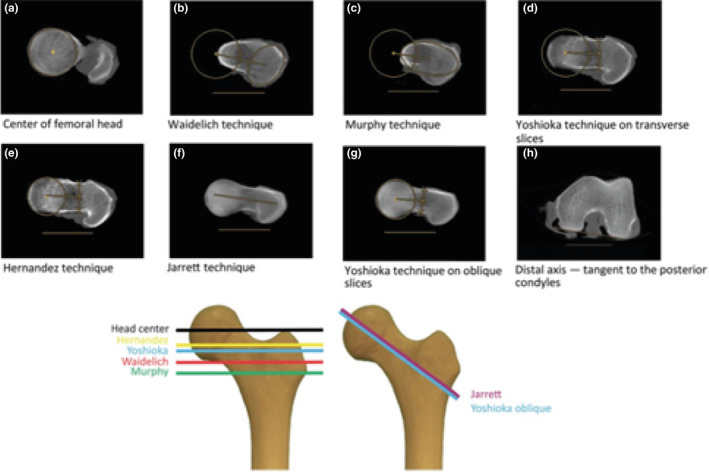
Top: Examples of different methods of femoral neck anteversion (FNA) assessment and how they affect the assessed geometry: A, B, C, D, E are transverse slice methods (Hernandez *et al.,* 1981; Murphy *et al.,* 1987; Yoshioka et al., 1987; Waidelich et al., 1992; Jarrett *et al.,* 2010), and F and G use oblique slices (Yoshioka *et al*., 1987; Jarrett *et al*., 2010). The location of the slices in the coronal plan is shown in the lower panel. H shows that the posterior condylar line was taken as reference for all methods. Figure from Kaiser *et al*. (2016). Below: left, the proximal and distal part of the femur is superimposed in this picture. The lines through the neck depict different neck axes and table top condylar axes looking along the shaft axis: ‘Neck’ refers to the Berryman method (Berryman *et al.,* 2014), a semiautomatic method taking into account the femoral head centre, the base of the femoral neck and the cluster of points of the neck. The Lee 2D (Lee *et al.,* 2006) method uses a straight line connecting the femoral head centre and the most cephalic junction of the greater trochanter on one axial slice. The Reikeras (Reikerås *et al.,* 1983) method uses a line connecting the centre of the femoral head on one slice and centre of the femoral neck on the slice that has the posterior and anterior edges of the neck running parallel. Murphy (Murphy *et al.,* 1987) uses a line connecting the femoral head centre on one axial slice and the centre of the base of the neck on another axial slices. Figure from Berryman *et al*. (2014). Right: column 1 axial slice cranial, column 2 axial slice through the neck centre, column 3 axial slice through the base of the neck with little head left. Row A neck axis defined as centre of femoral head and centre of femoral neck. Row B neck axis defined as line connecting the two centres of the width of the neck; row C above I is the line connecting the femoral head and the greater trochanter lateral edge, and the line below is the anterior border of the femoral neck as in ultrasound methods

There is evidence that femoral torsion occurs throughout the femoral shaft, below the lesser trochanter, and at the intertrochanteric level (Seitlinger *et al.,* 2016; Waisbrod *et al.,* 2017; Archibald *et al.,* 2019). As well as variation in clinical cases identified above, substantial variation in torsion within each of these regions has also been identified in non‐clinical populations (Seitlinger *et al.,* 2016; Ferlic *et al.,* 2018). FNA is considered the “total” femoral torsion. The definition of FNA and the chosen femoral axes determine the measurement. The femoral axes are defined as follows: the neck shaft axis, the femoral shaft axis and the condylar axis. The shape of the proximal part of the femur is complex, as the lateral part of the femoral neck is elliptical and its major axis tilts anteriorly (Backman, 1957), and the femoral head is not usually centred on the femoral shaft (Kingsley and Olmsted, 1948). The femoral neck axis can be defined as the line connecting the femoral head centre to the femoral shaft axis (Murphy *et al*., 1987; Waidelich *et al*., 1992), the line going through the centre of the femoral head to the narrowest part of the neck (Yoshioka *et al.,* 1987; Kim *et al.,* 2000b), the centre of the femoral neck (Reikerås *et al.,* 1983), the centre of the greater trochanter (Batailler *et al.,* 2018) or the edge of the greater trochanter (Sangeux *et al.,* 2015), the latter being referred to as a functional axis, as it takes into account the contact point of the hip and the insertion of the adductor muscles. The femoral neck axis could also be defined as the line parallel to the femoral neck (Weiner *et al.,* 1978; Wedge *et al.,* 1989), without taking into account the trochanter or the femoral head (Figure 7). These reconstructions are available in single cross‐sections of the femoral neck or on two different slices, further increasing differences even with similar definitions. For 3D models, the femoral neck axis may either be determined using a line of best fit of the centroids of the slices defining the neck as identified by hand (Sugano *et al.,* 1998a) or using principal component analysis on a point cloud covering the femoral neck determined semi‐automatically (Berryman *et al.,* 2014).

The anatomical femoral shaft axis is difficult to define accurately, due to the anterior curvature of the shaft. We can find its distal point at the anterolateral border of the posterior cruciate ligament (Yoshioka *et al.,* 1987), at the centre of the medial and lateral articular margins (Walmsley, 1933), at the midpoint of the centroids of each condyle (Berryman *et al.,* 2014), at the centre of the segment joining the two midpoints of the line connecting the anterior and posterior points of each condyle (Egund and Palmer, 1984), at the centroid of an axial cross‐section of the femoral condyle (Murphy *et al.,* 1987) or at the most proximal aspect of the intercondylar fossa (Sangeux *et al.,* 2015). The proximal point of the femoral axis has been defined using the centroid of the slice taken between the lesser trochanter and the greater trochanter (Murphy *et al.,* 1987; Buddenbrock *et al.,* 1997; Berryman *et al.,* 2014), under the lesser trochanter (Egund and Palmer, 1984; Sangeux *et al.,* 2015) or at the head centre if the functional weight‐bearing axis is used as the rotational axis (Yoshioka *et al.,* 1987). The femoral shaft axis in most cases is used as the rotation axis of the femur (Yoshioka *et al.,* 1987; Kim *et al.,* 2000b), as a fixed point to determine the neck axis (Murphy *et al.,* 1987; Buddenbrock *et al.,* 1997) or as a reference for femoral rotation (Egund and Palmer, 1984). In 3D models, it is possible to establish the anatomical femoral axis via the interpolation of the centroids of multiple slices taken along the femoral shaft (Eckhoff *et al.,* 2016).

**FIGURE 8:**
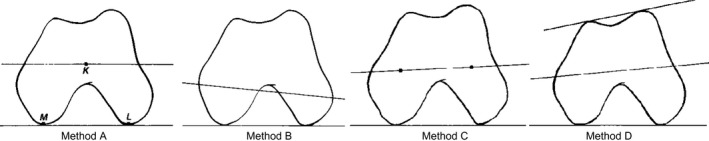
Different methods used to define the distal femoral axis. Method A (posterior condylar line), classical table top method, with posterior condyles lying on the table. Method B (epicondylar line), the most medial and lateral extremes of the condyles on the axial view (Weiner *et al.,* 1978). Method C identifies the centroids of the medial and lateral condyles. Method D bisects the angle formed by the posterior and anterior condylar lines. Figure from Murphy et al. (1987)

The distal femoral axis (Figure 8) has been defined in various ways: as the posterior edge of the lateral and the medial condyle (posterior condylar line)(Kingsley and Olmsted, 1948; Egund and Palmer, 1984; Murphy *et al.,* 1987; Berryman *et al.,* 2014; Sangeux *et al.,* 2015; Eckhoff *et al.,* 2016), the midline between the anterior condylar line and the posterior condylar line (Ruby *et al.,* 1979; Hernandez *et al.,* 1981), or as a line connecting the peak of the epicondyles on a transverse view (epicondylar line) (Weiner *et al.,* 1978; Yoshioka *et al.,* 1987). The posterior condylar line has been shown to be the least location‐dependent and the most repeatable method to define the distal femoral axis among those outlined (Murphy *et al.,* 1987). However, the most relevant axis from a biomechanical perspective remains to be determined. The functional distal axis has been proposed to lie along the epicondylar line, alternatively a variation of this axis using the sulcus of the medial epicondyles has been defined as the logical reference for rotation of the femoral component in knee arthroplasty (Griffin *et al.,* 2000).

## 7 MEASUREMENT OF FNA

### 7.1 Computed tomography (CT)

Computed tomography can be used to acquire images of single cross‐sections or volumes of bone. Because the X‐ray attenuation is very different between bone and soft tissue, CT provides a sharp contrast between bone and soft tissue and is therefore good in depicting mature, well‐ossified bones. CT scanning times per slice are lower than 2 s and for 3D‐rendering of hip structures, which requires multiple slices, this can go up to 40 s (Falchi and Rollandi, 2004). CT is considered cheap compared with MRI due to shorter scanning time. CT results in ionising radiation exposure of 0.3–0.5 mSv for six slices in adults (Muhamad *et al.,* 2012); this dose would be double in neonates (Brenner and Hall, 2007).

Single axial slices using CT can be used to define the neck axis (Weiner *et al.,* 1978; Hernandez *et al.,* 1981; Jend, 1986; Beebe *et al.,* 2017). However, the height (Jend, 1986; Sugano *et al.,* 1998a) and angle of slices used for the 3D reconstruction (Tomczak *et al.,* 1997; Schneider *et al.,* 1997; Beebe *et al.,* 2017) affect the final result, as the femoral neck has an asymmetrical shape (Backman, 1957). Alternatively, two axial slices can be taken to define the femoral neck axis, one at the femoral head and the other at different heights of the distal femoral neck (Egund and Palmer, 1984; Murphy *et al.,* 1987; Waidelich *et al.,* 1992; Buddenbrock *et al.,* 1997). Where scans are not aligned with the femoral shaft axis, calculation may be needed to transform the torsion to a reference plane, as positioning‐related measurement errors can be as large as 8.8° (Hermann and Egund, 1997). After 3D rendering of the proximal femur it is possible to take an oblique slice along the femoral neck angle in the coronal plane (Kim *et al.,* 2000b; Jarrett *et al.,* 2010) or compute the femoral neck axis in 3D (Sugano *et al.,* 1998a; Kim *et al.,* 2000a; Lee *et al.,* 2006; Berryman *et al.,* 2014). The distal femoral axis can be evaluated with one single axial slice with reference to one of the axes described earlier.

### 7.2 Magnetic resonance imaging (MRI)

Magnetic resonance imaging obtains similar features of the transverse femoral cross‐section to CT. MRI uses strong magnetic fields and radio waves to exploit paramagnetic properties, mostly of freely movable protons, to generate images and is therefore free of ionising radiation. Different from the bone approach with CT, which measures the presence of bone apatite, MRI measures the absence of freely movable protons in bone. Moreover, the MRI magnetic field often limits the application to subjects who do not have metal implants, pacemakers or other contraindications. In addition, undergoing an MRI measure of the femur means remaining still within a narrow, confining tube for between 5 (Koenig *et al.,* 2012) and 20 min (Tomczak *et al.,* 1995), depending on the sequence type, or up to 45 min where 3D rendering is required (Botser *et al.,* 2012), which limits its application in young children without sedation. Furthermore, MRI is usually expensive and not available in all research and clinical facilities. It has been suggested that MRI can be superior to CT in depicting the proximal and distal femoral contours in children with immature bones (Tomczak *et al.,* 1997; Rosskopf *et al.,* 2017). The orientation of scan slices is virtually equally achievable with CT and MRI (Koenig *et al.,* 2012; Rosskopf *et al.,* 2017; Beebe *et al.,* 2017) although alignment of CT scans requires post‐scan reconstruction. In contrast, MRI scans can be directly aligned to anatomical features such as the femoral neck (Tomczak *et al.,* 1995), thereby allowing the depiction of the whole region. This is particularly useful at high femoral neck inclination angles, where positioning bias is greatest (Jarrett *et al.,* 2010). The distal femoral axis can be evaluated with one single axial slice with reference to one of the axes described earlier.

### 7.3 Ultrasound imaging (US)

Ultrasound imaging with most clinical scanners only enables two‐dimensional cross‐sectional views of soft structures and only identifies the outer surface of mature (fully mineralised) bones. On the other hand, the imaging of whole cross‐sections is possible in neonates and young infants where the bone is not yet mineralised and remains permeable by sound waves. US is free of ionising radiation, cheap compared with other imaging techniques, and fast in terms of image acquisition, with the whole protocol lasting up to 10 min (Kulig *et al.,* 2010). Free‐hand ultrasound approaches use motion capture or other techniques to track the probe in 3D and are therefore more expensive and time‐consuming, taking between 8 and 15 min (Passmore *et al.,* 2016; Greatrex *et al.,* 2017) if only particular landmarks are of interest, as with FNA, or longer if a 3D model of the whole femur is required (Świątek‐Najwer *et al.,* 2014).

Some methods place the probe horizontally and measure the inclination on the image on the screen or later on the printed image Moulton and Upadhyay, 1982; Upadhyay *et al*., 1987; Elke *et al*., 1991) but results are not consistent at high angles of anteversion (Phillips *et al.,* 1985; Terjesen and Anda, 1987; Elke *et al.,* 1991) where the distal part of the femoral neck becomes deeper and harder to image. To adjust for this issue, others use inclinometers mounted on the probe (Terjesen and Anda, 1987; Terjesen *et al.,* 1993; Aamodt *et al.,* 1995; Ehrenstein *et al.,* 1999) and take the measurement when the chosen features are showing horizontally on the screen, or use additional hardware to place the femur in internal rotation (Elke *et al.,* 1991). Free‐hand US couples the ultrasound with video or motion capture localisers (Keppler *et al*., 1999; Keppler *et al*., 2007; Świątek‐Najwer *et al.,* 2014; Passmore *et al.,* 2016; Greatrex *et al.,* 2017).

**FIGURE 9:**
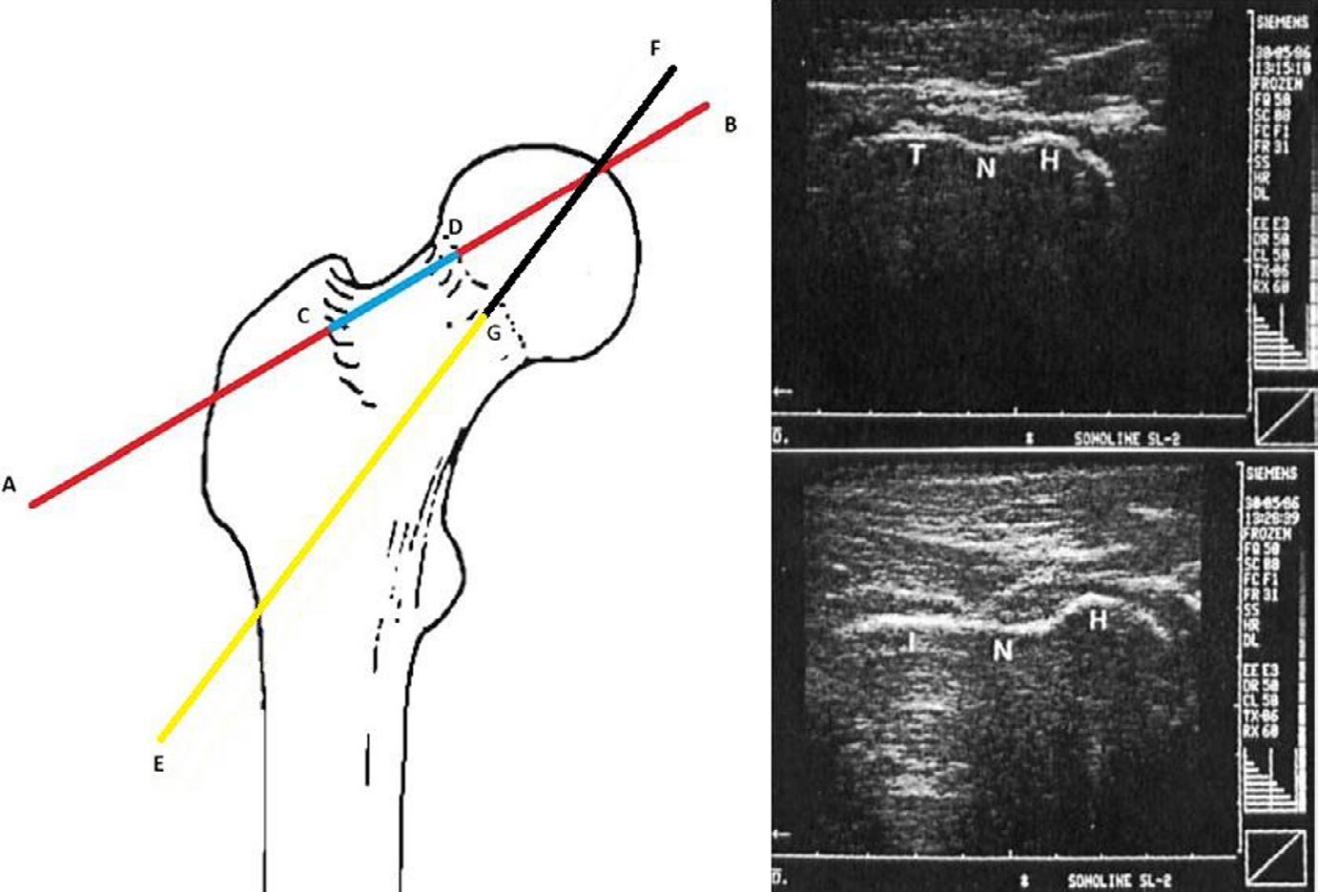
Left panel adapted from Elke *et al*. (1991). Frontal view of different ultrasound approaches: the head‐trochanter line approach features the peak of the red ‘head’ section BD and the peak of the red ‘trochanter’ section AC (Upadhyay et al., 1987; Terjesen and Anda, 1987; Aamodt *et al.,* 1995; Keppler *et al.,* 1999). The femoral neck approach assesses the region parallel to the blue CD section (Clarac *et al.,* 1985; Ehrenstein *et al.,* 1999). The intertrochanteric plane approach assesses the bone parallel to the yellow GE line (Elke *et al.,* 1991; Kulig *et al.,* 2010; Passmore *et al.,* 2016). Right panel: ultrasound images used to define FNA. H = femoral head, N = femoral neck, T = greater trochanter, I = intertrochanteric plane. The head‐trochanter line and parallel to the neck line can be drawn from the top right panel. The parallel to the intertrochanteric plane can be drawn from the bottom right panel. Figure from Terjesen *et al*. (1993)

Several features have been used to determine the proximal femur axis: the head‐trochanter tangent (Upadhyay et al., 1987; Aamodt *et al.,* 1995; Keppler *et al.,* 1999), the femoral neck (Clarac *et al.,* 1985; Ehrenstein *et al.,* 1999), and the intertrochanteric plane (Elke *et al.,* 1991; Kulig *et al.,* 2010; Passmore *et al.,* 2016) (Figure 9). However, taking only the anterior border into account means that inter‐individual differences in the angle between the anterior border and centre of the femoral neck are ignored. The features used to draw the inter‐condyle axis are the posterior condyles (Keppler *et al.,* 1999), the epicondyles (Moulton and Upadhyay, 1982, the epicondyles, and the anterior condyles (Upadhyay et al., 1987) or the anterior condyles only (Ehrenstein *et al.,* 1999). The posterior condylar line can also be inferred using the tibia as a perpendicular reference (Terjesen and Anda, 1987; Elke *et al.,* 1991; Terjesen *et al.,* 1993; Günther *et al.,* 1996), bearing in mind that varus or valgus knee deformation could affect the end result.

### 7.4 Radiography

The images yielded from radiography are projections of the bone structures in the space between the generator and the detector. The detection of the femoral neck axis can be done by single‐plane radiographs giving a 2D image (Dunn and Notley, 1952), biplanar radiography allowing two different projections (Dunlap *et al.,* 1953; Ryder and Crane, 1953; Rippstein, 1955; Magilligan, 1956; Lee *et al.,* 1992) or a 3D computer reconstruction (Chaibi *et al.,* 2012). The actual image acquisition takes seconds, whereas positioning depends on the participant but is usually 1–2 min (Rosskopf *et al.,* 2014). Analysis of single‐plane images involves drawing a line through the femoral neck axis and is therefore quick. In biplanar and 3D models, the post‐processing can take from 2 min for approaches where only gross features are identified, to 20 min for techniques requiring the identification of a large number of landmarks and evaluation of the whole lower limb (Rosskopf *et al.,* 2014). In the biplanar method, lines are drawn through the chosen landmarks and then converted using trigonometric conversion tables (Dunn and Notley, 1952; Dunlap et al., 1953; Ryder and Crane, 1953; Rippstein, 1955; Magilligan, 1956; Lee *et al.,* 1992). In 3D reconstructions, the images are evaluated by the software. The recently introduced EOS imaging technique (a low‐dose radiography system) semi‐automatically draws the silhouette of the bone and 3D models of the lower limb (Chaibi *et al.,* 2012).

Furthermore, radiography is cheap and available in most clinical facilities. The downside of the method is that it uses ionising radiation, reported to be in the range of 0.25 mSV for low‐dose radiography to 7.5 mSV for regular radiography (Kalifa *et al.,* 1998; Brenner and Hall, 2007).

Biplanar methods involve an initial anteroposterior radiograph in a supine (Ryder and Crane, 1953; Rippstein, 1955; Magilligan, 1956), prone (Dunlap *et al.,* 1953) or standing position (Lee *et al.,* 1992; Chaibi *et al.,* 2012). The second image is taken with the hip flexed at 90,° with various degrees of abduction for each method (Ryder and Crane, 1953; Dunlap et al., 1953; Rippstein, 1955; Ogata and Goldsand, 1979), with the detector parallel to the femoral neck inclination (Magilligan, 1956) or standing (Lee *et al.,* 1992; Chaibi *et al.,* 2012). In most methods, the proximal axis is taken as parallel to the femoral neck (Dunlap *et al.,* 1953; Rippstein, 1955; Magilligan, 1956; Ogata and Goldsand, 1979) but in others the femoral head‐trochanter line (Lee *et al.,* 1992; Chaibi *et al.,* 2012) or head‐neck centre is assessed (Ryder and Crane, 1953). The distal axis can be defined using the posterior condylar line (Chaibi *et al.,* 2012); alternatively, the tibia is considered perpendicular to the condylar line (Dunlap et al., 1953; Ryder and Crane, 1953; Rippstein, 1955; Magilligan, 1956; Ogata and Goldsand, 1979; Lee *et al.,* 1992).

### 7.5 Functional assessment

It is reported to be possible to measure the FNA without any imaging methods by assessing the angular range of motion (ROM) of the hip joint in the axial plane. FNA can be assessed using the ratio of internal rotation over external rotation, with greater internal rotation associated with greater FNA (Cibulka, 2004; Chadayammuri *et al.,* 2016). In this case, the assumption is that the end of the internal and external rotation gives information about where the femoral head stops gliding in the acetabulum and the femoral neck prevents further rotation by touching the contour of the acetabulum. Another way of measuring FNA is by measuring the angle of rotation at the point where the greater trochanter feels most prominent, via palpation of the lateral hip (Ruwe *et al.,* 1992; Davids *et al.,* 2002). In this case, the assumption is that when the trochanter is most lateral during the rotation of the femur, the femoral neck is parallel to the floor and the angle of the tibia will indicate the FNA. This method is called the trochanteric prominence angle test (TPAT), or Craig's test. The downside, however, is the accuracy and precision of these functional methods. These are cheap and convenient, requiring only a goniometer or camera to take measurements. These methods are indirect indicators of femoral version and are dependent on both capsular and muscular restraints, as well as acetabular version (Gelberman *et al.,* 1987; van Arkel *et al.,* 2015; Chadayammuri *et al.,* 2016).

The measurement of the rotation has been performed with both an extended and a flexed hip (Ruwe *et al.,* 1992; Davids *et al.,* 2002; Botser *et al.,* 2012; Kelly *et al.,* 2012; Chadayammuri *et al.,* 2016), and the degree of flexion can affect the measured FNA by 15° (Chadayammuri *et al.,* 2016). A hip flexion of 45° was used to yield intermediate results between 0° and 90° of flexion (Tönnis and Heinecke, 1991). It has been suggested that performing functional tests in extended and in flexed positions will give additional information on capsular restraint and acetabular involvement (Gelberman *et al.,* 1987; Cibulka, 2004; van Arkel *et al.,* 2015; Chadayammuri *et al.,* 2016). In all of these methods, the tibia is taken as the perpendicular reference of the posterior condyles (Gelberman *et al.,* 1987; Ruwe *et al.,* 1992; Chung *et al.,* 2010; Botser *et al.,* 2012; Sangeux *et al.,* 2014; Chadayammuri *et al.,* 2016; Uding *et al.,* 2019).

### 7.6 Differences between methods

FNA measurements are dependent on the imaging technique and the landmarks used (as listed in Table 1) with differences in mean values between methods of up to 10° (Kaiser *et al.,* 2016), which can increase to 20° when people with exaggerated anteversion are tested (Schmaranzer *et al.,* 2019). In a study comparing repeatability of measurements using different landmarks on the same CT scans, mean intra‐observer error was between 0.8° and 2.9° for the methods of Waidelich, Jarret, Yoshioka, Murphy and Hernandez, with a range up to 11.4° in Hernandez's method (Kaiser *et al.,* 2016). Interobserver repeatability is excellent for both oblique (interclass correlation ICC 0.95) (Buck *et al.,* 2012; Kaiser *et al.,* 2016; Beebe *et al.,* 2017) and axial CT techniques (ICC 0.87, 0.93–0.96) (Kaiser *et al.,* 2016; Beebe *et al.,* 2017; Schmaranzer *et al.,* 2019).

**FIGURE 10:**
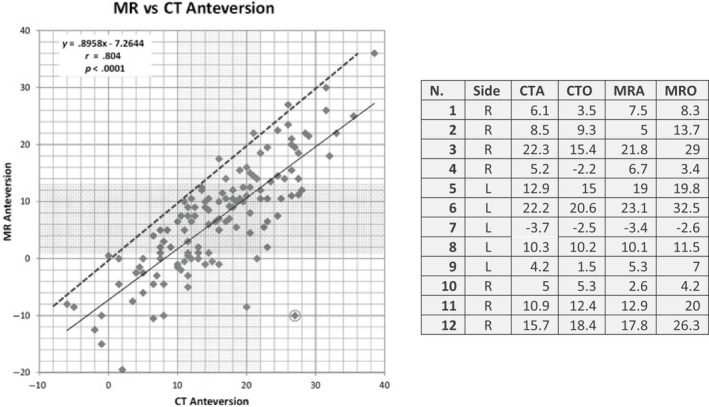
Left: comparison of oblique magnetic resonance imaging (MRI) and computer tomography (CT) measurements of the FNA. Figure modified from Botser *et al*. (2012). The vertical shaded area represents the middle two quartiles of the CT measurement and the horizontal shaded area represents the middle two quartiles of the MRI measurement. The circled data point is the one with the greatest discrepancy between CT and MRI FNA values. The solid line is the line of best fit and the dashed line is the identity line. Right: comparison of CTA (computer tomography axial), CTO (computer tomography oblique), MRA (MRI axial) and MRO (MRI oblique). Note the systematic difference between MRI and CT values in the left panel. In addition the large differences occur in same specimens using the same reference axes to measure FNA but with different imaging techniques (MRI and CT). Adapted from Beebe *et al*. (2017)

Inter‐ and intra‐operator reliability of MRI in the measurement of FNA has been shown to be comparable to CT (ICC 0.90‐0.97) (Tomczak *et al.,* 1997; Schneider *et al.,* 1997; Muhamad *et al.,* 2012; Beebe *et al.,* 2017). A number of studies have compared CT and MRI measures with different methods (Günther *et al.,* 1996; Schneider *et al.,* 1997; Tomczak *et al.,* 1997) and, in some cases, different landmarks (Kaiser *et al.,* 2016) or even different samples (Muhamad *et al.,* 2012). However, studies comparing the same methods, meaning the same slice height, orientation and landmark choice, with either MRI or CT show a systematic lower result of 8.9° in MRI with differences up to 37° (Figure 10 left) (Botser et al., 2012) or up to 13.6° variation between measures in another study (Figure 10, right) (Beebe *et al.,* 2017). It has been suggested that the reason for this systematic error might be the long scanning time of the MRI, which causes the subject to relax and change positions between the scanning of the proximal and distal femur. Alternatively, differences in the appearance of bone tissue between MRI and CT may lead to differences in the positioning of identified landmarks.

**FIGURE 11:**
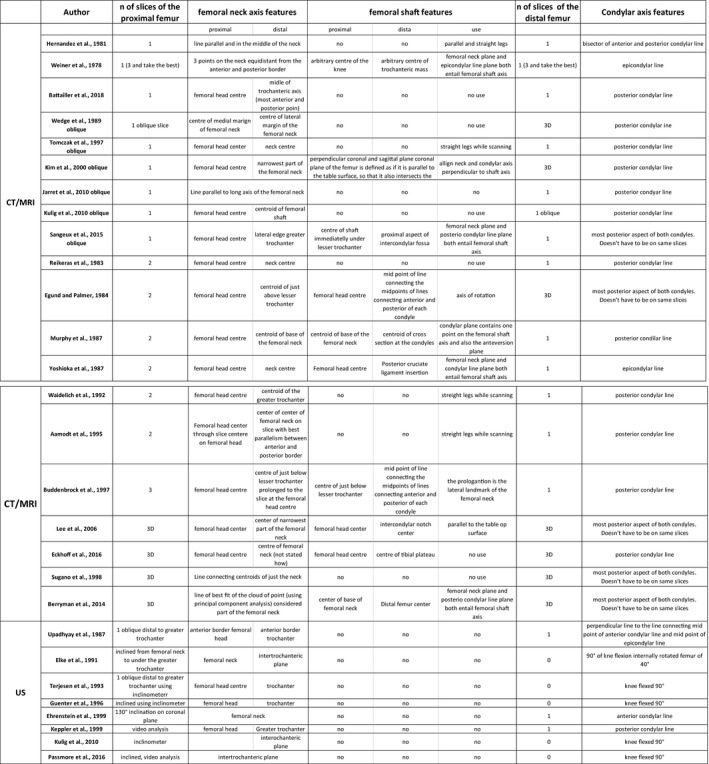
Current methods to measure femoral neck anteversion (FNA), with short explanation of landmarks used

Comparisons of US with different imaging techniques such as radiography, MRI and CT have shown mean differences of FNA of 0.5° to more than 10° (Terjesen and Anda, 1987; Upadhyay *et al*., 1987; Elke *et al*., 1991; Aamodt *et al*., 1995; Tomczak *et al*., 1995; Ehrenstein *et al*., 1999; Keppler *et al*., 1999; Kulig *et al*., 2010; Passmore *et al*., 2016). US has been shown to have lower inter‐ and intra‐observer reliability than MRI or CT for the 2D methods (Tomczak *et al.,* 1995) but appears more reliable in dried bones (Upadhyay et al., 1987). This may be due to the ability to detect landmarks directly on the dried bone visually, rather than relating them to images on screen through the soft tissue.

US has been considered a good screening method for FNA because of lower inter‐ and intra‐reliability or only moderate correlation (*r* = .57–.87) with other imaging techniques (Terjesen and Anda, 1987; Elke *et al.,* 1991; Tomczak *et al.,* 1995; Aamodt *et al.,* 1995) even though these discrepancies also result from errors in the compared methods. US results are closer to MRI than to CT (Tomczak *et al.,* 1995), which may be because the landmarks can be obtained in line with the inclination of the femoral neck. However, the free‐hand US methods appear more repeatable compared with regular US (Keppler *et al.,* 1999; Keppler *et al.,* 2007; Passmore *et al.,* 2016), with average errors as low as 1.8° (Passmore *et al.,* 2016) and MRI and ICC of 0.95 (Greatrex *et al.,* 2017).

We can find a total average difference for biplanar radiography compared with dried femoral measurements of 2.6°–3.6° with good correlation (*r* = .82–.91) (Dunlap *et al.,* 1953; Rippstein, 1955; Ogata and Goldsand, 1979; Lee et al., 1992). However, in living subjects, the error could be as high as 20° due to positioning errors (Wissing and Spira, 1986). The EOS system yields total average differences compared to CT of 0°–5° (Buck *et al.,* 2012; Rosskopf et al., 2014). Inter‐reader agreement is high for the low‐dose EOS system (ICC 0.95) with an average difference of 0.1°and 3.4° (Buck *et al.,* 2012; Rosskopf *et al.,* 2014). Error sources might include inaccurate positioning due to physical impairments (such as spasticity, pain, skeletal deformities or obesity) and inaccurate location of the axes on the roentgenogram (Gibson, 1967) because of lack of clear guidelines. Additionally, soft tissue can obscure the bone outline, making detection of the bony structure more difficult in obese populations.

Davids *et al*. (2002) have shown that even though the total mean difference of functional methods is below 5°, in 45% of the sample the error is more than 10° compared with CT. However, an internal rotation test or TPAT is good at predicting a low, normal or high anteversion (Kelly *et al.,* 2012; Muhamad et al., 2012; Chadayammuri *et al.,* 2016; Uding *et al.,* 2019). Correlations with CT methods are highly variable, with regression coefficients ranging from less than 0.25 to 0.79 (Chung *et al.,* 2010; Botser *et al.,* 2012; Sangeux *et al.,* 2014; Uding *et al.,* 2019) for the internal rotation test, and from 0.12 (Sangeux *et al.,* 2014) to 0.86 (Chung *et al.,* 2010; Uding *et al.,* 2019) for TPAT. The reliability of the method is good: the interobserver ICC value for internal ROM is 0.89 and for the TPAT it is 0.81 (Chung *et al.,* 2010).

## 8 CONCLUSIONS

Abnormal FNA changes the biomechanics of the hip, altering muscular lever arms, hip contact forces and femoral neck shear forces, which may contribute to development of a wide range of skeletal disorders, such as osteoarthritis and alter the kinematics of the lower limbs. FNA grows in line with the growth plate, which seems to adjust according to mechanical forces acting on the proximal femur during movement, increasing the FNA during gestation to more than 30° and thereafter decreasing steadily until completion of growth. This decrease is less pronounced or absent in individuals with conditions causing neuromuscular and movement impairment such as cerebral palsy. Interestingly, FNA in older adults is consistently lower than in younger adults, raising the question of mechanisms behind the modelling of the femur at a mature age following growth plate closure. Treatment for altered FNA is usually de‐rotational osteotomy, suggested only in the case of disabling conditions; passive, non‐operative methods such as braces or wearable cables do not have any effect. Despite observational evidence for the effects of muscular activity on FNA development during growth, the efficacy of targeted physical activity remains unexplored. Large variations between methods evaluating the FNA limit the ability to synthesise the large number of studies on the topic, as normative values must be set, relative to the method. It is not possible to draw conclusions on the choice of which femoral axis to utilise, and further studies are needed to determine the relevant forces shaping it. However, the authors endorse identification of landmarks which consider the femoral head and trochanter part of the femoral neck axis. Further studies are needed to explore the distal axis and whether the usual posterior condylar axis is the most relevant one. As for the imaging technique, it is situation‐driven, with MRI and CT giving the best images and measurement precision; MRI is superior to CT in terms of radiation hazard and CT is quicker and cheaper. The biplanar radiography EOS system seems to be a quick, low‐radiation option but it is not as reliable as the aforementioned approaches. However, both within clinical and basic science research, limitations of these methods prevent their broader application in healthy children and population studies. New US techniques already permit a 3D depiction of the femur with systems of probe localisation. Further improvements in US imaging and data analysis could provide a cheap, quick, non‐invasive and more broadly applicable alternative to MRI and CT.

## CONFLICT OF INTEREST

Matteo Scorcelletti, Neil Reeves, Jörn Rittweger and Alex Ireland declare that they have no conflict of interest.

## AUTHOR CONTRIBUTIONS

Conception or design of the work: Matteo Scorcelletti and Alex Ireland. Drafting the article: Matteo Scorcelletti. Critical revision of the article: all authors. Final approval of the version to be published: all authors.
